# DL3D: visual representation of conformational ensembles of domain-linker-domain proteins

**DOI:** 10.1186/s12859-026-06474-4

**Published:** 2026-05-29

**Authors:** Laure Carrière, Simon Bartels, Christophe Zanon, Pau Bernadó, Juan Cortés

**Affiliations:** 1 LAAS-CNRS, Université de Toulouse, CNRS, Toulouse, France; 2 Institut de Mathématiques de Toulouse, Université de Toulouse, CNRS, Toulouse, France; 3 Centre de Biologie Structurale, Université de Montpellier, INSERM and CNRS, Montpellier, France

**Keywords:** Protein dynamics, Intrinsically disordered regions, Flexible linkers, Conformational ensembles, 3D visualization

## Abstract

**Background:**

The domain-linker-domain (DLD) architecture is commonly found in proteins, where flexible linkers connect consecutive domains and regulate their relative spatial positioning. Often, these linkers present partially structured elements that modulate inter-domain dynamics, directly influencing their function. From a protein design perspective, tuning the relative position and orientation of domains via the linker offers opportunities to modulate biological activity. Despite their relevance, analyzing conformational ensembles of DLD proteins remains a challenge, thereby limiting the structural insights that can be extracted.

**Results:**

We present DL3D, a robotics-inspired visualization tool that enables intuitive analysis of the conformational space sampled by DLD proteins. DL3D discretizes the relative positions of the two domains at the linker ends and projects each conformation onto a 3D voxel map, where density is represented in grayscale to highlight the most probable configurations. In addition, quaternion-based operations allow the analysis of relative domain orientations.

**Conclusion:**

DL3D facilitates the structural investigation of highly flexible proteins composed of well-folded domains connected by flexible linkers. Beyond visualization, the tool supports downstream analyses such as low-dimensional conformational clustering. DL3D is implemented as a Python package and is available at: https://gitlab.laas.fr/moma/methods/analysis/dl3d/. A Jupyter notebook with usage examples is also provided.

## Background

The domain-linker-domain (DLD) architecture is a common structural pattern found across all kingdoms of life [10], with studies reporting that approximately 65% of eukaryotic proteins and 40% of prokaryotic proteins contain multiple domains [6]. In this arrangement, individual domains typically carry out distinct biological functions, such as catalysis, signaling, or molecular recognition, while the linker connecting them plays a crucial though often overlooked role in regulating their spatial and functional interplay [17].

Linkers are usually flexible, and often lack well-defined secondary structure. Their sequence tends to be poorly conserved, and they frequently exhibit intrinsic disorder [8]. Despite their apparent simplicity, linkers critically modulate protein behavior by determining the relative orientation and spacing between domains. For instance, in multi-domain enzymes, flexible linkers can spatially tether catalytic and binding domains, thereby increasing the local effective concentration of substrates and controlling inter-domain mobility to improve catalytic efficiency [19]. They are also involved in conformational regulation, including mechanisms like self-inhibition or allosteric activation driven by domain rearrangements [7, 11, 12].

While significant efforts in protein engineering have focused on the design of well-folded domains, the design of linkers remains largely empirical and underexploited [2, 8]. To better harness their functional potential, there is a growing need for tools that provide detailed structural insight into linker behavior. Traditional analysis of intrinsically disordered regions often reduces conformational data into one-dimensional descriptors, such as radius of gyration or end-to-end distance, sacrificing information about relative position and orientation. Few studies have attempted to capture richer spatial information for DLD proteins. For example, the disk-on-sphere method proposed by Qi et al. [18] uses directional statistics to represent the relative orientation between domains, but it does not simultaneously account for their positional relationships.

In this context, we present DL3D, a Python-based visualization tool designed to analyze conformational ensembles of DLD proteins. DL3D is inspired by robotics, where graphical 3D representations have been proposed to visualize the reachable workspace of a robotic arm, including position and orientation [20]. Drawing on robotic arm analogy, DL3D treats one domain as a fixed base and visualizes the motion of the second domain, connected via the flexible linker, within a voxelized 3D space. Each conformation is projected onto a voxel, and voxel density reflects the frequency of occurrence, highlighting the most probable spatial arrangements. To fully capture domain pose, DL3D also incorporates quaternion mathematics to compute and visualize relative domain orientations. This approach builds on earlier voxel-based visualization methods applied to molecules [3] and extends them to include orientation analysis for richer structural interpretation. By enabling intuitive, spatially resolved visualization of inter-domain motions, DL3D provides a useful framework for studying linker behavior and opens new avenues for rational design of multi-modular proteins.

**Fig. 1:**
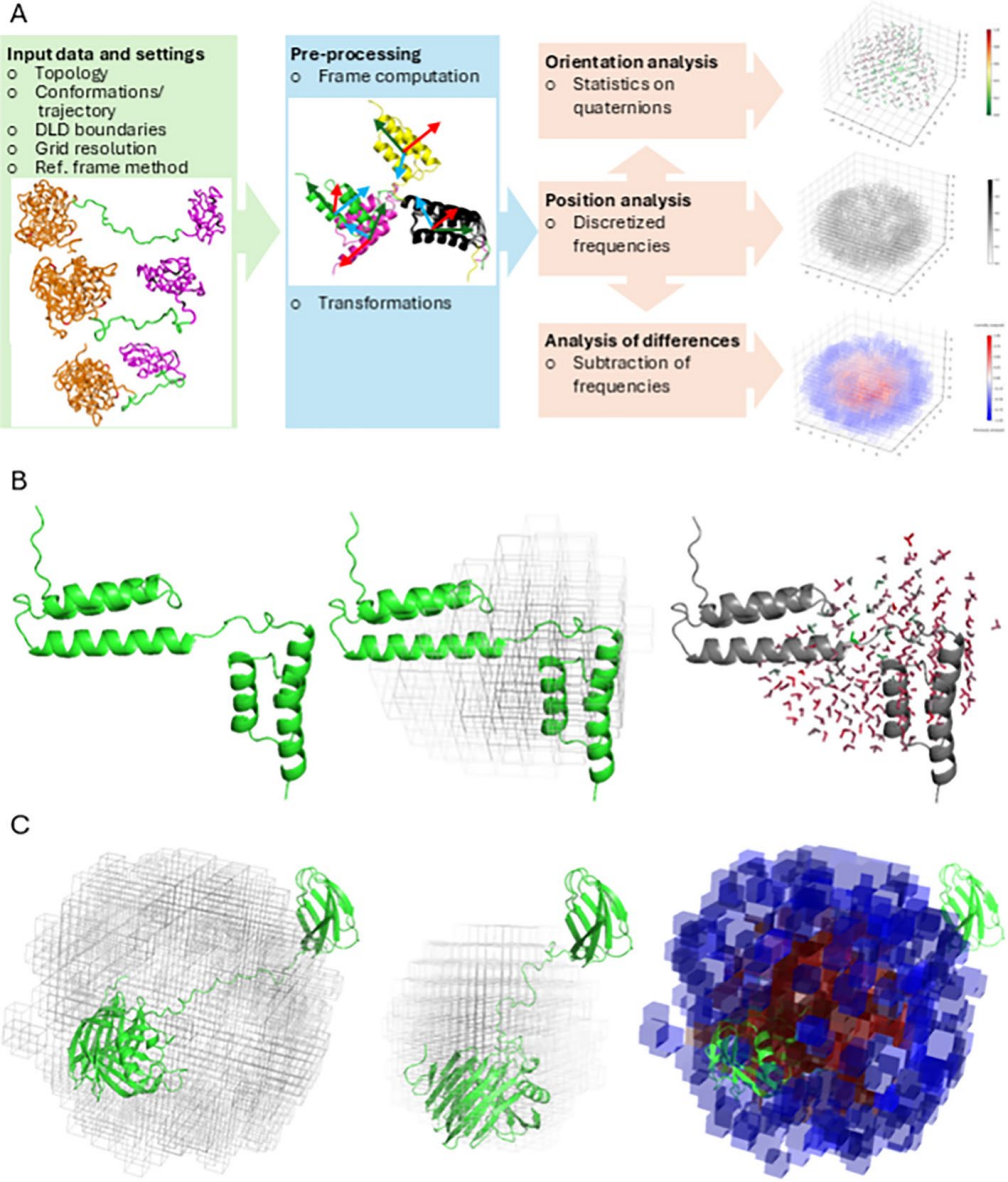
Overview of the DL3D method and applications. **A** Schematic overview of the DL3D workflow, comprising three main stages: (i) specification of input structures and analysis parameters, (ii) preprocessing of conformational ensembles, and (iii) voxel-based spatial and orientational analyses. Representative visual outputs are shown on the right. **B** Application of DL3D to ZLBT-C (left), previously studied by Qi et al. [18]. The voxel map reveals preferred relative positions of the two domains, highlighted by higher-density (darker) central voxels. The corresponding orientational analysis shows a preferred inter-domain orientation. Colors encode the variance of the orientations of frames assigned to each voxel. **C** Application of DL3D to a multi-modular enzyme. Voxel maps of the glycoside hydrolase (GH) domain positions with the carbohydrate-binding module (CBM) fixed in space, for two linker variants: a P-rich linker (left) and a G-rich linker (center). The right panel shows the voxel-wise difference between the two maps, highlighting distinct spatial distributions induced by linker composition. Red voxels indicate regions with a higher occupancy of the G-rich linker relative to the P-rich linker, whereas blue voxels indicate the opposite

## Implementation

The DL3D pipeline is illustrated in Fig. 1.A. The main stages are presented below.

### Input data and settings

DL3D requires as input a conformational ensemble and some parameters:

- *Conformational ensemble* Two types of input are supported: (i) a set of single conformations in PDB or mmCIF format, or (ii) a topology file (PDB or mmCIF) together with a trajectory file in any format supported by MDTraj [15]. Both, all-atom or coarse-grained (CG) models are supported. The files should contain only a single chain and no water molecules, ions or ligands. Importantly, the conformations are assumed to be physically realistic, with correct bond geometry and no steric clashes.
- *Reference frames* There are different options to define a reference frame associated to each domain. The method assumes that domains can be approximated as rigid bodies. Explanations about reference frames, which are the basis for the definition of relative poses, are provided below. The choice of the specific procedure used to compute the reference frames depends on the user’s preferences and on the intended downstream analyses. Nevertheless, the performance of DL3D is expected to be robust with respect to minor variations in the definition of these frames.
- *Type of analysis* This parameter refers to the different analyses that can be performed. In brief, DL3D enables visualization of the distribution of relative positions between the two domains, either with or without showing their average orientation. It also allows comparison between two ensembles to identify and analyze their differences.
- *Type of output* By default, DL3D produces a PyMol python script that can be further adapted by the user. Alternatively, DL3D can generate a matplotlib plot, either producing an image file or interactively embedded in an active figure. In addition, a human-readable JSON file is generated, enabling users to perform additional analyses from the voxel-based structuring of the conformational ensemble.
- *Grid resolution* size of the individual voxels.
- *Visualization parameters* By default, only the edges of the voxels are displayed to facilitate visualization of the interior of the voxel map. Alternatively, the voxel faces can be rendered with partial transparency. Weakly populated voxels are not shown. This can be controlled either by a threshold defined as a percentage of the ensemble size (5% by default) or by specifying a minimum number of conformations.

### Pre-processing

A Cartesian reference frame is computed for each domain in each conformation. Three options are available:

- The default option relies on selecting three amino acids (AAs) within each domain: one set for the fixed domain and one set for the mobile domain. These AAs define three reference points per domain, whose C*α * atom coordinates are used to construct the frame via the Gram–Schmidt process. The selected AAs may be consecutive (e.g., located at the beginning or end of a domain, near the linker) or chosen arbitrarily within the domain (for example, one near the center and two at different surface locations).
- When all-atom models are provided as input, the frames can be computed using only 1 AA in each domain. These AAs can be those connecting the domains to the linker, or can be chosen at a central location inside each domain. Each frame is then computed based on the coordinates of the N, C*α * and C atoms using the Gram–Schmidt process.
- The frames can also be computed using a larger set of amino acids. One may consider the entire domain or only a subset of residues, for example restricting the selection to one or several secondary structure elements. This approach is particularly useful when the domain is not perfectly rigid over the course of the trajectory. The location of the reference frame for a domain is defined as the center of mass of the given set of residues. Then the singular value decomposition of the matrix of differences between each residue to the center of mass is computed, and the resulting *3× 3* orthogonal matrix is used as basis. The motivation behind this protocol is that this matrix captures spatial properties of the provided residues: for example, for an *α *-helix, the first column corresponds to the axis of the helix [18].

Once the reference frames have been established, the relative pose (i.e., position and orientation) of the mobile domain with respect to the fixed domain for each conformation can be determined using operations on homogeneous transformation matrices [4].

### Position analysis

The distribution of the relative position of the mobile domain with respect to the fixed one can be represented as a point-cloud in $\mathbb {R}^3$. DL3D discretizes this space with a given resolution, thus producing a voxel-map. The intensity of each voxel is computed from the proportion of conformations that it contains.

### Orientation analysis

For all the conformations corresponding to each voxel, additional analyses can be performed to provide information about the preferred relative orientation of the domains and its variability. For each conformation, the rotation component of the homogeneous transformation matrix is converted into a quaternion $q_i$. The average quaternion $\bar{q}$ for a voxel is the eigenvector corresponding to the largest eigenvalue of $Q = \sum _{i=1}^n q_iq_i^T$ [13], and its variance $\sigma ^2 = \frac{1}{n}\sum _{i=1}^n d(q_i,\bar{q})^2$, where *d* is the angular distance between two unit quaternions. The average quaternion $\bar{q}$ is converted back into a rotation matrix, whose three orthogonal axes are visualized as cylinders. Variance is indicated by a color scale from green (low) to red (high).

### Analysis of differences

DL3D also allows users to visualize structural differences between conformational ensembles corresponding to different linkers, or between ensembles with the same linker generated by different methods or under different conditions. This option is also interesting to analyze differences between ensembles before and after refinement with respect to experimental data, such as Small-Angle X-ray Scattering measurements [1]. For this, users first need to perform an analysis for one of the ensembles with one of the methods described above. Then, given a second ensemble, this method generates a voxel-map where voxels are colored according to their relative difference in intensity (i.e. local density of conformations) between the two ensembles. Gray voxels represent regions in 3D space for which the two ensembles have roughly equal frequency. The redder a voxel, the higher the relative number of conformations of the second ensemble in that region compared to the first ensemble, and *vice versa* for blue voxels.

## Results and discussion

### Analysis of relative positions and orientations

To illustrate the usage of DL3D for representing a conformational ensemble of a DLD protein in terms of the relative position and orientation of its two domains, we selected a target from the CASP16 Conformational Ensembles Experiment [14]. In particular, ZLBT-C is a construct derived from protein A, a virulence factor of *Staphylococcus aureus* that binds mammalian antibodies and inhibits opsonization. The CASP16 challenge included this biologically relevant system to evaluate predictions of interdomain pose distributions in such multi-domain proteins. ZLBT-C consists of two small rigid domains connected by a short flexible linker. A model of the protein was predicted using AlphaFold2 [9], and it is dislayed in Fig. 1.B. Then, a conformational ensemble was first generated using AFflecto [16], considering eight flexible residues between the domains. The sampled structures were then subjected to independent short (20 ns) molecular dynamics (MD) simulations to further explore conformational variability. From the resulting ensemble, a random subset of 3,300 conformations was selected. Reference frames were defined by selecting one residue located at the center of each of the three helices in each domain, and the voxel resolution was set to 6 Å (default value). Using DL3D to analyze the conformational ensemble, the positional voxel representation shows a higher population density in the central region (Fig. 1.B), indicating that certain relative domain arrangements are more frequently sampled under the physical constraints of the model. In addition, orientation analysis reveals that, within some of these highly populated voxels, the orientation distributions display low variance, suggesting that specific orientations are consistently favored within this physically accessible region. The total computing time, including data loading, which constitutes the most time-consuming step, was approximately 4 s for the position analysis and 6 s for the orientation analysis when run on a standard laptop computer.

### Comparison of conformational ensembles

DL3D can also provide a 3D representation of the differences between two conformational ensembles. To illustrate this, we selected two DLD constructions involving the same domains but different linkers. These constructions are inspired by natural multi-modular enzymes, in which a catalytic domain, here a glycoside hydrolase (GH), is tethered to a carbohydrate-binding module (CBM). We extracted sequences of this type of protein from the CAZy database [5] and selected two linkers of identical length (22 residues) but contrasting amino acid compositions: a proline-rich (P-rich) linker and a glycine-rich (G-rich) linker. As in the previous example, we generated conformational ensembles using AlphaFold2 combined with AFflecto, but without relying on MD simulations in this case. 10,000 conformations were generated for each construct, and all of them were used as input for DL3D. In this case, the frames were defined using the last and first residues of the domains preceding and following the linker, respectively. Note that this choice has little impact on the results. The spatial resolution was kept at the default value of 6 Å. Owing to the larger size of the proteins and the ensembles with respect to the previous example, the computing time was longer, on the order of 5 min. The results of the position analysis, shown in Fig. 1.C (two images on the left), clearly indicate that the P-rich linker tends to position the two domains far apart, whereas the G-rich linker favors intermediate inter-domain distances. Scarcely populated voxels are omitted to improve clarity. Interpretation is further facilitated by analyzing the differences between voxel maps, shown in the right panel of the figure. Outer voxels display much higher intensities for the P-rich linker, whereas central voxels are more populated for the G-rich linker.

Overall, DL3D provides an efficient and intuitive framework for visualizing the conformational ensembles of DLD proteins and for assessing their relative domain positions and orientations in light of experimental observations. These results demonstrate the value of DL3D as an accessible visual tool for interpreting structural differences arising from linker composition or environmental conditions, and highlight its potential for guiding rational linker design.

## Conclusions

DL3D provides an efficient and intuitive framework to visualize conformational ensembles of DLD proteins generated from molecular dynamics simulations or other modeling approaches, allowing a direct assessment of relative domain positions and orientations. The method can also be combined with experimental restraints, such as those derived from SAXS measurements, to support ensemble refinement and facilitate the interpretation of low-resolution scattering data. By enabling straightforward comparisons between ensembles obtained under different conditions or with different linker compositions, DL3D helps identify structural trends and highlight key determinants of interdomain flexibility, thereby supporting the rational design and optimization of linker regions.

DL3D is not intended as a replacement for dimensionality-reduction techniques or other specialized tools for the analysis of MD trajectories, but rather as a complementary and more geometrically interpretable representation of conformational ensembles.

Beyond DLD proteins, DL3D could be extended to the analysis of protein complex ensembles generated by docking or structure prediction methods, by treating individual chains or subunits as rigid bodies. This would provide a natural framework to interpret relative poses and orientations in multi-component assemblies.

Another possible extension concerns other biomolecular systems such as RNA, where reference frames could be defined for selected structural motifs in order to analyze their relative poses, even when these regions are only approximately rigid.

## Data Availability

Data analyzed in the current study are available at https://moma.laas.fr/static/data/DL3D/Top_P_rich.pdb (P-rich topology file), https://moma.laas.fr/static/data/DL3D/Top_G_rich.pdb (G-rich topology file), https://moma.laas.fr/static/data/DL3D/Traj_P_rich.xtc (P-rich trajectory file) and https://moma.laas.fr/static/data/DL3D/Traj_G_rich.xtc (G-rich trajectory file) for the CAZy proteins and at https://moma.laas.fr/static/data/DL3D/Top_CASP.gro (topology file) and https://moma.laas.fr/static/data/DL3D/Traj_CASP.xtc (trajectory file) for the ZLBT-C case. Due to its large size, only part of the ZLBT-C dataset is provided; the complete dataset is available from the corresponding author upon request. The Jupyter notebook enables users to reproduce the results by supplying the datasets and parameters directly.

## Abbreviations

DLD: Domain-Linker-Domain
GH: Glycoside Hydrolase
CBM: Carbohydrate-Binding Module
CG: Coarse-Grained
AAs: Amino Acids
MD: Molecular Dynamics
